# Safety and efficacy of emergency transarterial embolization for mesenteric bleeding

**DOI:** 10.1186/s42155-021-00281-z

**Published:** 2022-01-08

**Authors:** Chloé Extrat, Sylvain Grange, Clément Chevalier, Nicolas Williet, Jean-Marc Phelip, Fabrice-Guy Barral, Bertrand Le Roy, Rémi Grange

**Affiliations:** 1 Department of Radiology, University Hospital of Saint-Etienne, Avenue Albert Raimond, 42270 Saint-Priest-En-Jarez, France; 2 Department of Gastro-Enterology, University Hospital of Saint-Etienne, Saint-Priest-En-Jarez, France; 3 Department of Digestive and oncologic surgery, University Hospital of Saint-Etienne, Saint-Priest-En-Jarez, France

**Keywords:** Mesentery, Hematoma, Embolization, Bleeding

## Abstract

**Background:**

Patients with spontaneous or traumatic active mesenteric bleeding cannot be treated endoscopically. Transarterial embolization can serve as a potential alternative to emergency surgery. Literature on transarterial embolization for mesenteric bleeding remains very scarce. The objective of this study was to evaluate the safety and efficacy of transarterial embolization for mesenteric bleeding. We reviewed all consecutive patients admitted for mesenteric bleeding to the interventional radiology department, in a tertiary center, between January 2010 and March 2021. Mesenteric bleeding was defined as mesenteric hematoma and contrast extravasation and/or pseudoaneurysm visible on pre-operative CT scan. We evaluated technical success, clinical success, and complications.

**Results:**

Among the 17 patients admitted to the interventional department for mesenteric bleeding, 15 presented with active mesenteric bleeding requiring transarterial embolization with five patients with hemodynamic instability. Mean age was 67 ± 14 years, including 12 (70.6%) males. Technical success was achieved in 14/15 (93.3%) patients. One patient with technical failure was treated by percutaneous embolization with NBCA-Lipiodol mixture. Three patients (20%) had early rebleeding: two were treated by successful repeat embolization and one by surgery. One patient (6.7%) had early death within 30 days and two patients (13.3%) had late death after 30 days. Mean length of hospitalization was 12.8 ± 7 days. There were no transarterial embolization-related ischemic complications.

**Conclusion:**

Transarterial embolization is a safe and effective technique for treating mesenteric bleeding even in patients with hemodynamic instability. Transarterial embolization doesn’t close the door to surgery and could be proposed as first intention in case of mesenteric bleeding.

## Introduction

Mesenteric bleeding (MB) occurs rarely (Aoki et al., 1990) and its frequency is not well known. It corresponds to bleeding from mesenteric vessels in the abdominal cavity, without intra-luminal digestive bleeding. Although relatively rare, this pathology can be life-threatening if left undiagnosed and untreated. Clinically, MB are characterized by non-systematised abdominal pain and sudden blood loss. MB has many causes such as a post operative complication (especially after pancreaticoduodenectomy), traumatism, tumour, or may be idiopathic with no cause found. CT-scan is the gold standard of diagnostic imaging to identify the cause of MB (Brofman et al., 2006). While the management of upper and lower gastrointestinal bleeding has been well established (Chaudhary & Stanley, 2019; Gralnek et al., 2015; Barkun et al., 2019), the management of active mesenteric bleeding is less defined in the medical literature. Emergency laparotomy is indicated to drain a mesenteric hematoma, especially if it is associated with blunt bowel injury. However, surgery is not appropriate to localize and stop active MB. (Ghelfi et al., 2016). Thus, the question of whether to operate or not to operate upon patients with MB remains an open subject for discussion (Ghelfi et al., 2016). Unlike gastrointestinal bleeding, endoscopy is not suitable for treating mesenteric bleeding. Transarterial embolization (TAE) can therefore be considered as an alternative treatment modality. TAE is valuable in identifying the specific bleeding site and achieving haemostasis by elective exclusion of the bleeding artery using temporary agents (gelatine sponge slurry) or permanent agents (coils, NBCA, micro-particles). However, it poses the potential risk of digestive ischemia (Nykänen et al., 2018). TAE has been reported to be effective in the treatment of gastrointestinal bleeding (Bua-ngam et al., 2017; Bond & Smith, 2019; Beggs et al., 2014), and predictive factors for rebleeding and death after TAE have been reported in this indication (Choi et al., 2020). Endovascular treatment of mesenteric hematoma has been reported in a case report (Watanabe et al., 2021). Nevertheless, while only two studies have focused on post-traumatic MB (Ghelfi et al., 2016; Shin et al., 2016), no studies have investigated the efficacy of TAE in treating traumatic and spontaneous MB. The purpose of this study is therefore to report the safety and efficacy of emergency TAE for the treatment of mesenteric bleeding in a tertiary center.

## Material and methods

### Study population

We retrospectively reviewed all patients referred to our hospital for MB who were treated by TAE, based on clinical decisions in emergency and computed tomography (CT) images, between January 2010 and March 2021. MB was defined as mesenteric hematoma and contrast extravasation and/or pseudoaneurysm visible on pre-operative CT scan.

The inclusion criteria were as follows: (1) age 18 or over (2) patients suffering from acute arterial isolated MB and treated by TAE (3) active bleeding and/or pseudoaneurym on preoperative CT scan. Our exclusion criteria were (1) patients with no available pre-operative CT scan, (2) patients without available biological data, (3) patients with isolated retroperitoneal bleeding (4) patients with both mesenteric and gastro intestinal bleeding.

### Clinical data

We retrospectively reviewed clinical presentations at baseline, CT findings, cause of bleeding details of the embolization procedure, such as the angiographic findings and embolic materials used, procedure-related complications, and outcomes after TAE, including technical and clinical success and 30-day mortality rates. Patients who met one of the following criteria at patients’ initial presentation were classified in the coagulopathy group: international normalized ratio ≥ 1.5, platelet count less than 80G/L, and prothrombin ratio ≤ 50%. Hemodynamic instability was defined as PAS < 90 mmHg and/or a decreased in systolic pressure despite pharmacological support.

All patients underwent an abdominal CT scan (SOMATOM SENSATION before September and SOMATOM Siemens AG, Medical solutions, Erlangen, Germany). An unenhanced CT scan was first performed, followed by at least arterial and parenchymal phase and sometimes delayed phase. Patients received ≥90 ml contrast medium (Xenetix 350®, Guerbet, Villepinte, France) with a flow rate ≥ 3 ml/s.

### Angiography and embolization procedure

Angiographies and embolizations were performed by 8 interventional radiologists whom experience in TAE ranged from 2 to 30 years, after decisions made with the surgeon and the clinician. After local anaesthesia with 5 ml of Lidocaïne, the most common approach was through the right femoral artery. Celiac, superior mesenteric, and/or inferior mesenteric angiograms were performed to determine the focus of mesenteric injury using a 4F catheter and a hydrophilic guidewire (Terumo, Tokyo, Japan). All procedures were performed using fluoroscopy and/or roadmap technique. Supraselective catheterism was systematically performed, using a 2.7F microcatheter (Progreat, Terumo, Tokyo, Japan). The choice of embolic agent depended on the presence of pseudoaneurysm and/or active bleeding, the presence of collaterals, the location of the feeding artery, the clinical condition of the patient, and the habits of the physician. Embolization as close as possible to the point of bleeding was routinely performed to limit the risk of bowel ischemia. In case of visible collaterals, the coil trapping embolization technique using coils was performed. In case of embolization with NBCA, the microcatheter was flushed with 5% dextrose solution followed by injection of NBCA in a solution of ethiodized oil (Lipiodol, Guerbet, Villepinte, France) with 1/3 proportion. Embolizations were performed using fibered microcoils (Interlock Boston scientific, MA, USA), N-butyl-2-cyanoacrylate (NBCA)(Glubran)(Glubran2®, GEM, Viareggio, Italy), gelatin sponge (Gelitaspon®, Gelita Medical GmbH, Eberbach, Germany) or microparticles (Embosphere® Microspheres, BioSphere Medical, Rockland, MA). After the procedure, complete angiograms were performed to confirm that bleeding had been successfully controlled.

### Patient follow-up

After TAE, all patients were monitored closely for clinical signs and symptoms that were potentially suggestive of ischemic complication or recurrent bleeding until discharge or death. These clinical findings were supplemented by laboratory studies. The long-term outcome of the patients, specifically incidence of rebleeding, mortality and procedure-related complications were determined by chart review. CT scan following embolization was not a routine practice in the hospital unit during this period.

### Study endpoints

Technical success was defined as the cessation of angiographic extravasation, the absence of pseudoaneurysm filling immediately after embolization, based on the angiographic findings. Clinical success was defined as no rebleeding within 30 days after TAE or recurrence of bleeding that required only medical treatment or repeat TAE. Clinical failure was defined as haemorrhagic related-death or rebleeding that required emergency surgery during the 30-day follow-up.

Rebleeding events were classified as early events if they occurred within 30-days of embolization and as late rebleeding events if they occurred after 30 days of embolization.

Complications were defined as complications during the TAE and post-procedure complications during the follow-up period. Complications have been classified as minor or major according to the Clavien-Dindo classification (Dindo et al., 2004; GmbH A, n.d.). Grade I and II complications do not require treatment or only medical treatment and are classified as minor complications. Grade III, IV and V complications require endoscopic or surgical treatment, are life-threatening or result in death, and are classified as major complications.

### Statistical analysis

Qualitative variables are expressed as frequencies and percentages and quantitative variables, as means and standard deviation. Statistical analyses were conducted in STATA software.

### Ethical considerations

This study was approved by the Ethic committee of our institute (IRBN112021).

## Results

### Patients background

**Fig. 1:**
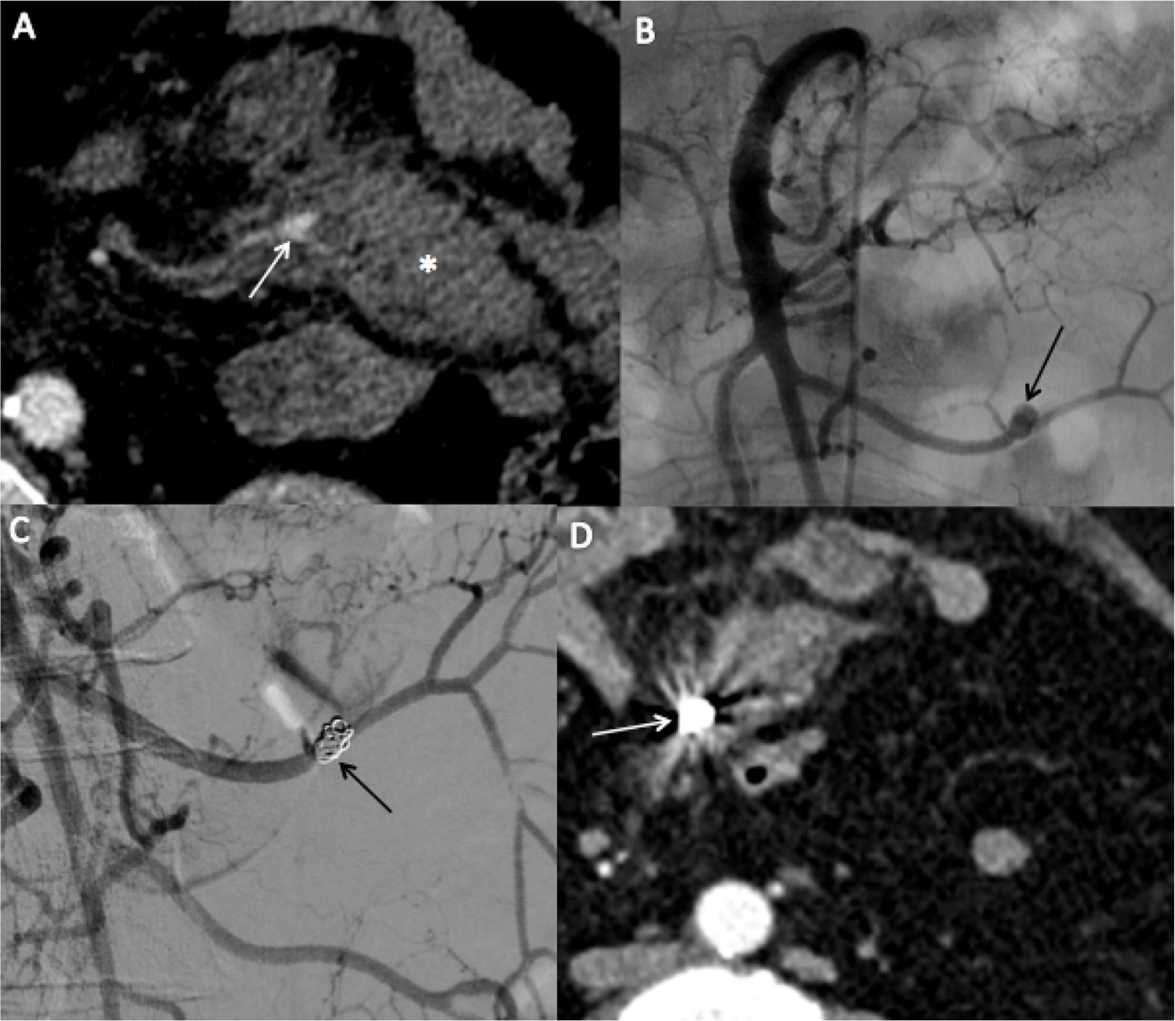
A 67-year-old female with blunt trauma. Axial CT scan (**A**) shows a mesenteric haematoma (*) associated with irregularity of a branch of the superior mesenteric artery, suggestive of pseudoaneurysm (white arrow). Angiography after catheterization of the SMA (**B**) confirmed the presence of a pseudoaneurysm (black arrow). After TAE using two fibered coils, the pseudoaneurysm is no longer filled (black arrow), and the feeding artery remains patent (**C**). A follow-up CT scan performed two days after embolization confirms the absence of filling of the pseudoaneurysm (white arrow) and the reduction in size of the haematoma (**D**)

Between January 2010 and March 2021, 788 emergency TAE have been performed in 743 patients in our institute. Seventeen patients were admitted to our interventional department for active MB requiring embolization. Demographic and clinical data are summarized in Table 1. The mean age was 67.4 ± 14.2 years old including 12(70.6%) males. All patients had pre-operative contrast-enhanced CT scan. Among the study population, 5 patients presented a hemodynamic instability and 2 patients had a coagulopathy with PR ≤ 50% and INR ≥ 1.5. Causes of bleeding were post-operative (5/17 patients), median arcuate ligament (3/17), pancreatitis (2/17), traumatic (2/17) (Fig. 1), idiopathic (2/17), pancreatic tumour (1/17), gastric tumour (1/17) and tumour adenopathy (1/17), On pre-procedure CT scans, contrast extravasation was noted in 7/17(41.2%) patients, pseudoaneurysm in 7/17(41.2%) patients, and both contrast extravasation and pseudoaneurysm in 3/17(17.6%) patients. For postoperative MB, the mean time between surgery and TAE was 10.0 ± 7.6 days. No MB was treated with surgery before embolization.

**Table 1:** Demographic data of the study population

Number of patients	17
**Age (years)**	
Mean ± SD	67.4 ± 14.2
**Male n. (%)**	12 (70.6)
**Previous disease n. (%)**	
Hyper Blood Pressure	11 (64.7)
Active cancer	6 (35.3)
Chronic renal failure	1 (5.9)
**Anticoagulation n. (%)**	6 (35.3)
**Trouble of haemostasis n. (%)**	
Prothombin ratio ≤ 50%	2 (11.8)
INR ≥1,5	2 (11.8)
**Preoperative CT scan n. (%)**	
Preoperative blush	7 (41.2)
Preoperative pseudoaneurysm	7 (41.2)
Both	3 (17.6)
**Nadir of Haemoglobin (g/dl)**	
Mean ± SD	9.2 ± 1.4
**Red Blood Cell transfusion n. (%)**	13 (76.4)
**Hemodynamic instability n. (%)**	5 (29.4)
**Etiology n. (%)**	
Post- operative	5 (29.4)
Traumatic	2 (11.8)
Pancreatitis	2 (11.8)
Tumor	3 (17.6)
Median arcuate ligament	3 (17.6)

### Angiography

Angiographic and outcomes data are summarized in Table 2. Two arteriograms in two patients with hemodynamic instability did not confirm active bleeding, resulting in 15/17 (88.2%) being treated by TAE. There was no empirical embolization. Among these two patients, one was immediately transferred to the operating room, because of significant hemodynamic instability and acute liver failure resulting in coagulopathy. Explorative laparotomy confirmed the hematomas which was spontaneously stopped. No active bleeding was seen either in arteriography or in surgery. He was discharged from hospital seven days later. The other patient was stabilized with conservative treatment and did not require surgical treatment.

**Table 2:** Procedure and outcomes for patients treated by TAE

Number of patients	15
**Localisation of bleeding n. (%)**	
Left gastric artery	2 (13.3)
Pancreaticoduodenal artery	4 (26.7)
Right gastroepiploic artery	2 (13.3)
Upper Mesenteric	4 (26.7)
Splenic artery	2 (13.3)
Dorsal pancreatic artery	1 (6.7)
**Material of embolization n. (%)**	
Coils	8 (53.3)
NBCA	3 (20)
Microparticles	1 (6.7)
Plug	1 (6.7)
Gelatine sponge slurry	1 (6.7)
Coils + Microparticles	1 (6.7)
**Duration of procedure (min)** Mean ± SD	92.9 ± 43.7
**Technical success n. (%)**	14 (93.3)
**Clinical success n. (%)**	14 (93.3)
**Per operative complication n. (%)**	0 (0)
**Post-operative complication n. (%)**	
Minor	0 (0)
Major	0 (0)
**Death n. (%)**	3 (20)
Early ≤30 days	1 (6.7)
Tardive > 30 days	2 (13.3)
**Recurrence of bleeding n. (%)**	
Early ≤30 days	3 (20)
Late > 30 days	0 (0)

Embolized arteries were pancreaticoduodenal (4/15), superior mesenteric (4/15), left gastric (2/15),, right gastroepiploic (2/15), splenic (2/15) and dorsal pancreatic artery (1/15). Eight patients were treated with microcoils, three with NBCA-Lipiodol mixture (1/3 ratio), one with microparticles, one with plug, one with both coils and micro-particles, and one with resorbable gelatin sponge. Among the eight patients treated with coils, four were treated by coils trapping, two by end-artery embolization and two by direct embolization of the pseudoaneurysm.

### Post angiography course

**Fig. 2:**
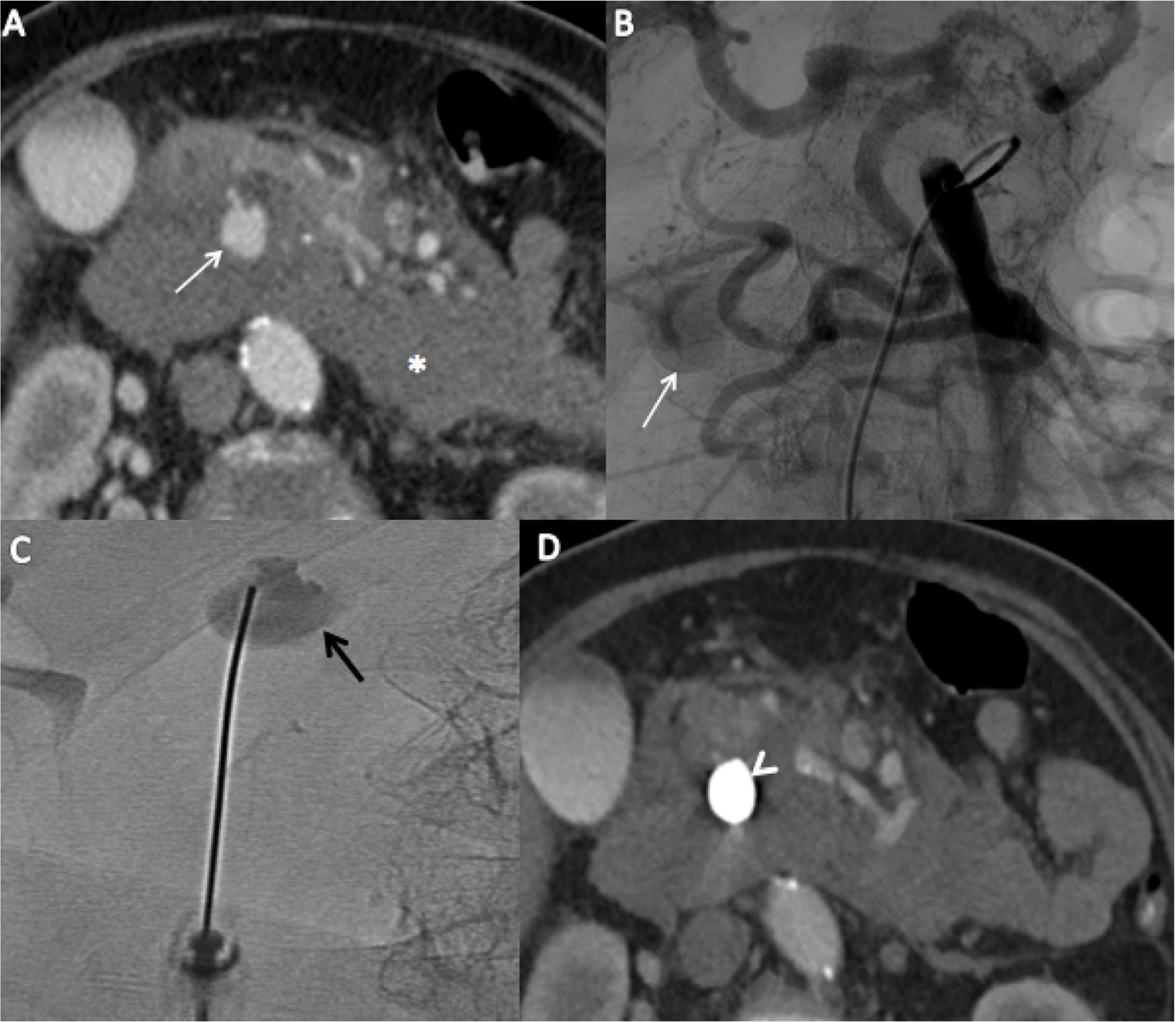
A 74-year-old female with spontaneous mesenteric haematoma following a median arcuate ligament syndrome. Axial CT scan (**A**) showed a pseudoaneurysm without contrast extravasation of a pancreaticoduodenal artery, with mesenteric hematoma (*). Upper mesenteric angiography (**B**) showed a prominent gastroduodenal artery that feeds the common hepatic artery and confirmed a pseudoaneurysm of 9 mm, without possibility to catheterize the feeding artery (endovascular technical failure). After puncture under ultrasonographic guidance, a fluoroscopic image (**C**) showed filling of the pseudoaneurysm using contrast agent injection (black arrow) with a 22G-needle. Post embolization contrast-enhanced axial CT scan (**D**) showed the absence of filling of the pseudoaneurysm (white arrow’s head)

Technical success was achieved in 14/15 (93.3%) patients. The only technical failure of TAE concerned a pseudoaneurysm of a pancreaticoduodenal artery in a 74-year-old female patient, which was not accessible via an endovascular approach. It was instead treated with a percutaneous approach, under ultrasonographic and fluoroscopic guidance, using NBCA-Lipiodol mixture (Fig. 2). Clinical success was achieved in 14/15 (93.3%) patients. The clinical failure concerned an 83-year-old male patient treated by bowel resection for occlusive syndrome with digestive necrosis complicated by a mesenteric hematoma. He underwent emergency embolization of the superior mesenteric artery using gelatine sponge. The CT scan performed 3 days later showed an increase in size of the mesenteric hematoma without active bleeding. The patient was taken to the operating room for surgical haemostasis and drainage of the hematoma. Three patients (20%) had early rebleeding: two were treated by successful repeat TAE and have been considered as clinical success. One was treated by surgery.

**Fig. 3:**
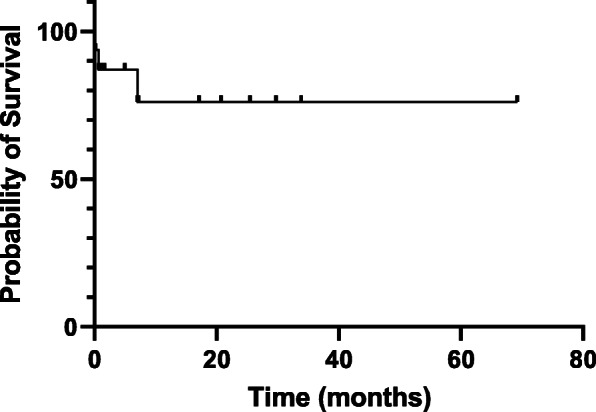
Kaplan Meier curve of overall survival of the 15 patients tretaed by TAE for mesenteric bleeding

No acute renal failure was reported after intra-arterial injection of iodinated contrast agents. There were no TAE-related ischemic complications such as bowel ischemia. One patient (6.7%) died within 30 days following TAE (Fig. 3). This 83-year-old male patient with gastric cancer and haemodynamic instability, died from acute respiratory distress syndrome, 4 days after initial successful embolization of tumour gastric bleeding. Of the remaining two patients with hemodynamic instability treated by TAE, neither had a recurrence of bleeding. Both of them are still alive. Of the two patients with coagulopathy, one was treated by TAE after receiving parenteral administration of 2 mg Vitamin K, with technical and clinical success. Two patients (13.3%) died at distance from embolization: one 7 months after the procedure of metastatic progression of an urothelial carcinoma and the other from a long chronic illness more than 4 years later. The mean length of hospital stay was 12.8 ± 7 days. Nine (53%) patients were hospitalized in intensive care.

## Discussion

The present study showed that TAE is safe and effective to treat active MB, with high technical and clinical success rates (93% and 93%, respectively) and no major or minor TAE-related ischemic complication.

Data in the literature are scarce regarding the place of TAE in the management of both non-traumatic and traumatic MB. To our knowledge, this is the first study of first-line TAE to treat both traumatic and non-traumatic mesenteric bleeding. Indeed, efficacy of TAE in blunt mesenteric haematomas has been shown in some case reports (Hagiwara & Takasu, 2009) and two retrospective study (Ghelfi et al., 2016; Shin et al., 2016). Moreover, it’s the first study to report TAE in case of hemodynamic instability. This management of TAE in case of hemodynamic instability is unusual but has been performed after discussion with surgeons. Their argument was that these haemorrhages were difficult to control intraoperatively with a high risk of grossly ligating the vessels resulting in postoperative digestive ischemia. Ghelfi et al. (Ghelfi et al., 2016) in a study including 7 patients reported that TAE of a MB following blunt abdominal trauma was feasible, with a high clinical success rate (85.7%) and an acceptable complication rate (28.6%). In this study, only two cases of mesenteric bleeding were traumatic, successfully treated by TAE (Fig. 1). This finding supports that trauma-related mesenteric haematomas can be treated by embolization as a first-line treatment in selective patients.

Angiography showed a high detection rate of the bleeding focus in patients with mesenteric bleeding. Thus, 2 patients did not required TAE, as no pseudoaneurysm or contrast extravasation was seen on angiography. Empirical embolization could have been an option for these two patients. In fact, empirical TAE has been proposed to treat obscure tumour bleeding, in the absence of active bleeding or pseudoaneurysm on angiography (Lee et al., 2020; Arrayeh et al., 2012). This practice can be effective in cases of hemodynamic instability as a last option treatment. The good clinical outcome of the patient without TAE and with conservative treatment leads us to believe that the wait-and-see attitude is also an option to consider.

This study shows one case of technical failure in a patient with a ruptured pseudoaneurysm of a duodeno-pancreatic arch, with failed celiac trunk catheterisation. Opacification of superior mesenteric artery showed a tortuous course through the duodeno-pancreatic arches, and the absence of feeding artery. A percutaneous embolization, using a fine 22G-needle and NBCA-Lipiodol mixture was performed (Fig. 2). Won et Al. (Won et al., 2015) showed that NBCA-Lipiodol mixture is safe and effective to treat visceral pseudoaneurysms by TAE. Previous retrospective studies (Yadav et al., 2016; Lal et al., 2020) and case reports (Marra et al., 2019; Griviau et al., 2018) (Grange et al., 2021) have reported that percutaneous approaches using a fine 22G-needle are safe and effective in treating parietal and visceral pseudoaneurysm, particularly when the feeding artery is not visible during fluoroscopic angiography.

Among all the MB (*n* = 17) in this study, 3 (17.6%) were spontaneous on rupture of pseudoaneurysm due to a median arcuate ligament syndrome (MALS). Symptomatic MALS is a rare condition, and the clinical significance of celiac artery compression is unclear. Bleeding following a pseudoaneurysm does not seem to be rare in this condition. TAE should be considered in these patients (Watanabe et al., 2021), even if the celiac artery may be difficult to catheterised. However, embolization only treats the symptomatic consequences but does not treat the cause. Laparoscopic median arcuate ligament release is the standard option to treat the cause of pseudoaneurysm formation.

Interestingly, no symptomatic bowel ischemia occurred within our study sample.

These results are in line with Ghelfi et Al. (Ghelfi et al., 2016) who reported only one ischemic bowel complication related to TAE. Moreover, the same study suggests that TAE may be safer for patients with superior mesenteric injuries because of the development of collateral vessels. Indeed, a majority of TAE in this study involved pancreaticoduodenal arteries or branches from the superior mesenteric artery, which are well established to be safe owing to the richness of collateral bed. These arteries have a more developed anastomotic network. None of the patients had branches of the inferior mesenteric artery, which has a less developed anastomotic network and are more susceptible to bowel ischemia (Nykänen et al., 2018; Lee et al., 2020; Kwon et al., 2019).

This study has some limitations. First, the retrospective data collection from a single institution may have selection bias of the patient cohort. Second, embolization related adverse events such as bowel infarction were evaluated by clinical records only and may underestimate the ischemic effects of TAE. Also, the small number of cases treated and the absence of a control group limited validity of the results.

## Conclusion

TAE is a potentially effective technique for treating mesenteric bleeding, including in cases of hemodynamic instability, and could be considered as the first approach. As in this study, surgery can be performed after up-front TAE in case of technical failure. Further studies should be carried out to assess the predictive factors for technical and clinical failure, and to evaluate which patients may benefit from this type of treatment.

## Data Availability

Data sharing is not applicable to this article as no datasets were generated or analysed during the current study.

## Abbreviations

MB: Mesenteric bleeding
CT: Computed Tomography
TAE: Transarterial Embolization
NBCA: N-Butyl Cyanoacrylate
